# NETosis is critical in patients with severe community-acquired pneumonia

**DOI:** 10.3389/fimmu.2022.1051140

**Published:** 2022-11-15

**Authors:** Yiming Zhang, Yan Li, Na Sun, Hanqi Tang, Jun Ye, Yang Liu, Quan He, Yangyang Fu, Huadong Zhu, Chengyu Jiang, Jun Xu

**Affiliations:** ^1^ Emergency Department, State Key Laboratory of Complex Severe and Rare Diseases, Peking Union Medical College Hospital, Chinese Academy of Medical Science and Peking Union Medical College, Beijing, China; ^2^ State Key Laboratory of Medical Molecular Biology, Department of Biochemistry, Institute of Basic Medical Sciences, Chinese Academy of Medical Sciences, Peking Union Medical College, Beijing, China

**Keywords:** NETosis, severe community acquired pneumonia, transcriptional read-through, lncRNA, RNA sequencing

## Abstract

Pneumonia is the fourth leading cause of death globally, and the reason for the high mortality rate of patients with severe community-acquired pneumonia (SCAP) remains elusive. Corticosteroid treatment reduces mortality in adults with SCAP but can cause numerous adverse events. Therefore, novel therapeutic targets need to be explored and new adjunctive immune drugs are urgently required. We analyzed the transcriptome data of peripheral blood leukocytes from patients with SCAP and healthy controls from three perspectives: differentially expressed genes, predicted functions of differentially expressed long non-coding RNAs, and transcriptional read-through. We discovered that the NETosis pathway was top-ranked in patients with SCAP caused by diverse kinds of pathogens. This provides a potential therapeutic strategy for treating patients. Furthermore, we calculated the correlation between the expression of genes involved in NETosis and the ratio of arterial oxygen partial pressure to fractional inspired oxygen. We identified four novel potential therapeutic targets for NETosis in patients with SCAP, including H4C15, H3-5, DNASE1, and PRKCB. In addition, a higher occurrence of transcriptional read-through is associated with a worse outcome in patients with SCAP, which probably can explain the high mortality rate of patients with SCAP.

## Introduction

According to the report on the global burden of diseases and injuries, the number of global deaths caused by pneumonia has decreased by nearly 500,000 compared to data acquired in 2000, and pneumonia remains the fourth leading cause of death globally (1). Substantial progress has been made in studying molecular mechanisms of pneumonia, such as inflammation and immunity, especially since the start of the Coronavirus Disease 2019 (COVID-19) pandemic. For example, the frequency of Th17 cells and the level of interleukin 17 (IL-17) are increased in the blood of patients with pneumonia (2, 3) and pneumococcal carriage (4), thereby enhancing innate immunity against pathogens by activating neutrophils and strengthening the inflammatory response. IL-1 can assist in host defense against lung infection by various pathogens (5–8), but excessive levels of IL-1 are harmful; therefore, blocking IL -1 is beneficial to patients with severe pneumonia (9). Despite continuous progress in characterizing pneumonia mechanisms, the poor prognosis of patients with severe pneumonia remains significant, and the reason for the high mortality rate of patients with severe community-acquired pneumonia (SCAP) remains elusive. Corticosteroid treatment reduces mortality in adults with SCAP (10), suggesting that restricting rampant inflammation is critical for patients with severe pneumonia. However, corticosteroid therapy can cause many adverse events, and the clinical benefits of other adjunctive immune therapies remain unclear (11). Therefore, novel adjunctive immune drugs are urgently required.

Here, we analyzed the transcriptome data of peripheral blood leukocytes from patients with SCAP and healthy controls to explore potential therapeutic targets from three perspectives: differentially expressed genes (DEGs), predicted functions of differentially expressed long non-coding (lnc)RNAs (DELs), and gene pairs with transcriptional read-through (TRT). TRT is a phenomenon in which transcripts extend beyond the transcription termination site of genes under diverse cellular stresses, such as viral infection, cancer, heat shock, oxidative stress, and hyperosmotic stress (12–15). Readthrough transcription can disrupt the 3D structure of a genome by decompacting chromatin, which can switch local chromatin from a transcriptionally inactive compartment to a transcriptionally active compartment (16). TRT can occur in two modes: upstream and downstream genes on the same DNA strand (cis-TRT) or different DNA strands (trans-TRT) (17).

Based on our transcriptome analyses, we discovered that NETosis is critical in patients with SCAP caused by diverse kinds of pathogens. NETosis is the process by which activated neutrophils produce and release neutrophil extracellular traps (NETs) (18). NETs mediate host defense by trapping and killing bacteria, fungi, and viruses (19). While NETs provide a robust defense against pathogens, increasing evidence indicates that they can cause tissue damage (20, 21). Because of its double-edged sword effect, we believe modulating NETosis is crucial to balance the immune system disorder in patients with SCAP. Therefore, we explored potential therapeutic targets for NETosis and found that *H4C15*, *H3-5*, *DNASE1*, and *PRKCB* could be novel potential therapeutic targets, thereby providing a new perspective on drug development.

## Materials and methods

### Participants

We selected 56 patients diagnosed with SCAP according to the American Thoracic Society and Infectious Disease Society of America 2007 guidelines (22) from the emergency department of Peking Union Medical College Hospital, Beijing, China. Clinical data and blood samples were collected on the first and second days of admission. Thirty-seven patients (66.1%) had identified pathogens, and 13 of 37 patients were infected with more than one pathogen (**Supplementary Table 1**). The identified pathogens included influenza A, cytomegalovirus, Epstein-Barr virus, adenovirus, rhinovirus, *Streptococcus pneumoniae*, *Klebsiella pneumoniae*, *Pseudomonas aeruginosa*, *Nocardia asteroides*, *Burkholderia cepacia*, *Mycoplasma pneumoniae*, *Candida albicans*, *Aspergillus fumigatus*, and *Pneumocystis carinii*. In this study, the overall mortality rate was 26.8% (**Table 1**). Healthy controls (defined as having no diagnosed disease and no inflammation) were also recruited from the emergency department of Peking Union Medical College Hospital (**Supplementary Table 2**). This study was approved by the Ethics Committee of the Peking Union Medical College (S500). Written informed consent was obtained from all patients.

**Table 1:** Baseline characteristics of patients with SCAP.

	SCAP
**Basic information**	
Sex:	
Female	18 (32.1%)
Male	38 (67.9%)
Age	45.5 [33.2;65.0]
Prognosis:	
Death	15 (26.8%)
Remission	41 (73.2%)
Pathogen:	
Non-virus	34 (60.7%)
Virus	22 (39.3%)
AKI:	
No	38 (67.9%)
Yes	18 (32.1%)
Fever duration (days)	7.00 [4.00;11.2]
FiO_2_ (percent)	50.0 [37.0;80.0]
PaO_2_/FiO_2_	157 [95.6;236]
**Complete blood count**	
WBC (10^9^ cells/L)	10.0 [7.10;14.1]
RBC (10^12^ cells/L)	4.00 [3.35;4.35]
NEUT%	87.2 [81.7;92.0]
LY%	8.05 [4.32;15.4]
PLT (10^9^ cells/L)	162 [107;248]
HGB (g/L)	118 [104;132]
HCT	0.34 [0.30;0.39]
**Laboratory examination**	
ALT (U/L)	26.0 [16.5;45.5]
Alb (g/L)	28.0 [25.0;31.0]
Tbil (umol/L)	10.1 [8.00;14.6]
Dbil (umol/L)	4.65 [2.88;7.23]
Cr (umol/L)	69.5 [53.0;98.0]
BUN (mmol/L)	6.19 [4.83;9.66]
Na (mmol/L)	137 [134;139]
K (mmol/L)	3.75 [3.50;4.20]
Glucose (mmol/L)	8.00 [6.45;11.7]
**Arterial Blood Gas**	
pH	7.43 [7.38;7.45]
PaCO_2_ (mmHg)	36.2 [31.7;39.9]
PaO_2_ (mmHg)	76.3 [64.9;90.0]
HCO_3_^-^ (mmol/L)	22.2 [19.8;24.9]
ABE (mmol/L)	-0.95 [-4.05;0.92]
Lac (mmol/L)	1.45 [1.10;1.90]

Categorical data are displayed as n (%), and continuous variables are shown as median [interquartile range]. AKI, acute kidney injury; FiO_2_, fraction of inspired oxygen; PaO_2_, partial pressure of oxygen; WBC, white blood cell count; RBC, red blood cell count; NEUT%, percentage of neutrophils; LY%, percentage of lymphocytes; PLT, platelet; HGB, hemoglobin; HCT, hematocrit; ALT, alanine aminotransferase; Alb, albumin; Tbil, total Bilirubin; Dbil, direct Bilirubin; Cr, creatinine; BUN, blood urea nitrogen (urea); Na, sodium; K, potassium; PaCO_2_, partial pressure of carbon dioxide; HCO_3_^-^, bicarbonate; ABE, actual base excess; Lac, lactic acid. Detailed information about the patients is provided in **Supplementary Table 1**. There is a missing value in ALT data.

### RNA sample preparation

Whole blood was centrifuged at 2,000 *g* for 5–10 min at 25°C. The pelleted blood cells were mixed with 10 mL red blood cell lysis buffer (R1010, Solarbio) for 5min on ice, and the mixture was centrifuged at 2,000–3,000 ×*g* for 5min at 4°C. The supernatant was discarded, and 5ml red blood cell lysis buffer was added to resuspend the precipitate. The resuspended solution was centrifuged at 2,000–3,000 ×*g* for 5 min at 4°C. The supernatant was discarded. Finally, 1.5 mL RNA later (76544, Qiagen) was added to the total RNA precipitate for RNA library preparation.

### RNA library preparation and RNA sequencing

Strand-specific libraries were generated using the NEBNext^®^ Ultra™ RNA Library Prep Kit for Illumina^®^ (NEB, USA) following the manufacturer’s recommendations. The library was sequenced on a Novaseq 6000 platform (NEB, USA), and 150 bp strand-specific paired-end reads were generated.

### Differential expression analysis

Clean reads with adapters and low-quality reads removed were aligned to the human genome reference sequence hg19 using Tophat2 (version 2.0.13) (23) for RNA-seq and WGCNA analyses. Clean reads were aligned to the GRCh38 reference sequence using HISAT2 (version 2.1.0) (24) for lncRNA analysis. Gene annotation and quantification were performed using featureCounts (version 2.0.1) (25). The Combat-seq function in the sva package was used to remove batch effects. DEGs and DELs were identified using the DESeq2 package (version 1.34.0) (26) in R (version 4.1.2). Independent hypothesis weighting (IHW, version 1.22.0) was used to correct multiple hypothesis testing (27). Genes with adjusted P-value < 0.05 and FC > 5 or < 0.2 were identified as DEGs. lncRNAs with adjusted P-value < 0.05 and FC > 10 or < 0.1 were identified as DELs.

### Prediction of novel lncRNA genes

*De novo* transcript assembly for each sample was performed using StringTie (version 1.3.6). The assembled transcripts were merged into a single file using the merge function in StringTie. Transcripts that met all of the following criteria were considered to be novel lncRNAs: 1) class code was “i”, “u”, “x”, “j” or “o” after comparison with reference annotation (Ensemble GRCh38.104) using GffCompare (version 0.11.2) (28); 2) transcript length was at least 200 bp; 3) transcript FPKM > 0.5 and transcripts per million (TPM) > 3; and 4) transcripts were non-coding. The coding potential of transcripts was tested by CPC2, CPAT, CNCI, and Pfam, and only the transcripts that were determined as noncoding in all tests were kept (**Supplementary Figure 1A**).

### DELs target gene prediction

The protein-coding genes adjacent to the DELs (50 kb upstream and downstream on the same chromosome) were screened as cis-acting target genes of DELs. Trans-acting target genes were predicted by 1) co-expression relationship between DELs and protein-coding genes (Spearman’s |R|>0.9, P-value<0.05), and 2) the potential of lncRNAs to bind protein-coding genes. Triplex Domain Finder (Version 0.13.2) was used to predict whether lncRNAs could bind protein-coding genes (29).

### Transcriptional read-through

The four-step screening was used to determine the occurrence of TRT. First, 11,784 adjacent gene pairs on the same strand (cis-TRT) and 5051 adjacent gene pairs on different strands (trans-TRT) were selected. The coverage tool in bedtools (version 2.23.0) was used to quantify the coverage of gene pairs and corresponding intergenic regions. Second, gene pairs with upstream-gene FPKM above the 25th percentile were screened for further analysis and visualization because TRT tends to occur at the end of actively expressed genes (16). Third, the FC of intergenic FPKM > 5 was set as the threshold to ensure that the transcript of the upstream gene was beyond the transcription termination site (TTS) in patients with SCAP. Fourth, gene pairs with patient-specific TRT were determined if the downstream-gene FPKM of patients was greater than 1.5 when the mean downstream-gene FPKM was 0 in control or the FC was greater than 1.5 when the mean downstream-gene FPKM was not 0. The purpose of the last step was to keep gene pairs in which TRT affected the transcription of the downstream gene.

### Weighted gene co-expression network analysis

The WGCNA package (version 1.70-3) on the R platform was used to perform a weighted gene co-expression network analysis.

### Enrichment and protein-protein interaction network analyses

Pathway and gene ontology (GO) enrichment analyses were performed using the MetaCore database (Clarivate Analytics, https://portal.genego.com/). Cytoscape (version 3.8.0) and Enrichment map (version 3.3.0) were used for biological process network visualization. A protein-protein interaction network of DEGs was built using the STRING website (https://string-db.org/) with a confidence score threshold of 0.9 and plotted with Cytoscape.

### Statistics

All statistical analyses were performed using R (version 4.1.2). Correlations were evaluated using Spearman’s correlation coefficient, except for WGCNA, for which Pearson’s correlation coefficient was used by default. The Wilcoxon rank-sum test was used to compare the number of gene pairs with TRT and principal component 1 of NETosis genes between different groups. The comparison between the pathogen groups and prognosis groups was performed using chi-square test. Statistical significance was set at P-value< 0.05.

## Results

### RNA-seq analysis reveals that NETosis is involved in SCAP

**Figure 1:**
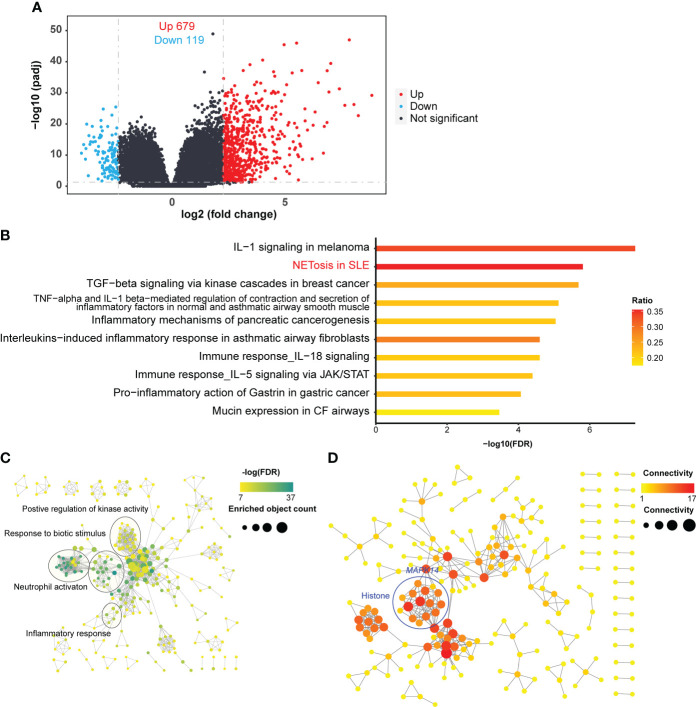
DEG identification and analyses. **(A)** Volcano plot of RNA-seq. The horizontal dashed line represents the P-value cutoff (0.05) for the differentially expressed genes (DEGs). The vertical dashed line represents the fold change (FC) cutoff of DEGs in patients with SCAP relative to healthy controls of DEGs (5 and 0.2). **(B)** Pathway enrichment of DEGs. The ratio indicates the proportion of the number of enriched network objects in a pathway to the total number of network objects in the pathway. **(C)** Gene Ontology Biological Processes enrichment map. The connection between biological processes is based on shared objects. **(D)** Protein-protein interaction (PPI) network of DEGs. Edge thickness indicates the strength of data support. Disconnected node is hidden in the network.

To explore potential therapeutic targets of SCAP, we analyzed RNA-seq data from peripheral blood leukocytes of patients with SCAP and healthy controls. We identified 798 significant DEGs (**Figure 1A**), of which 679 were upregulated (fold-change (FC) > 5, adjusted P-value < 0.05) and 119 were downregulated (FC < 0.2, adjusted P-value < 0.05).

We clustered DEGs using the MetaCore website to identify the biological processes and pathways in which they were involved. Of the top ten DEGs enriched pathways with a false discovery rate (FDR) < 0.05, most were related to immune or inflammatory responses, and the most significant pathway was IL-1 related signaling pathway (**Figure 1B**). The role of IL-1 is relatively well studied in infectious lung diseases (5–9), and numerous IL-1-targeting agents have been developed, some of which are approved for use (30–32). Therefore, we focused on the second most enriched pathway, NETosis in SLE, which is the only NETosis-related pathway in the MetaCore database. The NETosis-related cluster of biological processes included responses to biotic stimuli, inflammatory responses, neutrophil activation, and the positive regulation of kinase activity (**Figure 1C**). Responses to biotic stimuli, inflammatory responses, and neutrophil activation are prerequisites for NETosis, and the positive regulation of kinase activity may be related to kinase activation in NETosis (33).

We then sought to identify the genes crucial for NETosis in SCAP. We then performed a protein-protein interaction network analysis using the list of DEGs. Of the genes in the NETosis pathway, the histone-related genes (circled in blue, **Figure 1D**) and *MAPK14* were highly connected in the protein-protein interaction network (**Figure 1D**), suggesting that these genes are crucial for NETosis in SCAP.

### Weighted gene co-expression network analysis indicates that NETosis is correlated with the percentage of neutrophils

**Figure 2:**
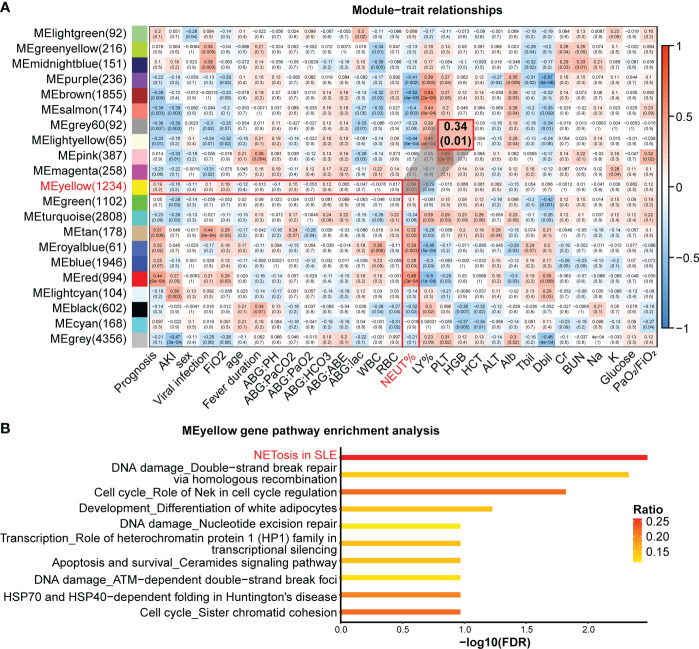
WGCNA of the RNA-seq. **(A)** Correlation heatmap of gene modules and clinical traits. Numbers in each cell represent Pearson’s correlation coefficients and the corresponding P-value (in brackets). **(B)** Pathway enrichment in the MEyellow gene module. The ratio indicates the proportion of the number of enriched network objects in a pathway to the total number of network objects in the pathway.

To explore the relationship between NETosis and clinical traits, we used weighted gene co-expression network analysis (WGCNA) to cluster genes and calculated the Pearson correlation coefficient between gene clusters and clinical traits (**Figure 2A**). We used the MetaCore database to enrich gene clusters, which are also called gene modules. The NETosis pathway was most significantly enriched (FDR = 0.003) in the MEyellow gene modules (**Figure 2B**). Therefore, we considered this gene module the most relevant to NETosis. We then examined the correlation between the MEyellow gene module and clinical traits. The MEyellow gene module showed the strongest correlation with the percentage of neutrophils (Pearson’s R = 0.34, P-value = 0.01; **Figure 2A**). This correlation suggests that NETosis is more likely to occur in patients with SCAP who have a higher percentage of neutrophils, which is consistent with the cellular mechanism of NETosis (33).

### NETosis may relate to viral infection, acute kidney injury, and higher partial pressure of carbon dioxide

**Figure 3:**
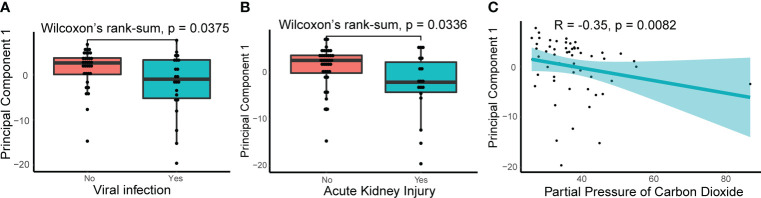
Relationships between principle component 1 of NETosis genes and clinical traits. **(A)** The principle component 1(PC1) of NETosis genes in patients with or without viral infection. The lines in the box-and-whisker plots represent the median Spearman’s correlation coefficient and the 0.25 and 0.75 quantiles. **(B)** The PC1 of NETosis genes in patients with or without acute kidney injury. **(C)** Spearman’s correlation between the PC1 of NETosis genes and partial pressure of carbon dioxide. The light-colored area indicates the confidence interval.

To explore the relationship between NETosis and clinical traits, we performed a principal component analysis on the expression levels of NETosis genes expressed in more than 50% of patients and extracted its principal component 1 (PC1). The correlations between the PC1 and original data were computed, and 107 out of 115 selected NETosis genes negatively correlated with PC1 (**Supplementary Table 3**). This indicates that PC1 can approximately represent how active the process NETosis is. The smaller the value of PC1, the more active NETosis is likely to be. We then explored the relationship between PC1 and clinical traits (**Supplementary Table 4**). PC1 was lower in patients with viral infections and acute kidney injury (**Figures 3A, B**). Furthermore, PC1 negatively correlated with partial pressure of carbon dioxide (**Figure 3C**), suggesting more active NETosis was associated with poor ventilation function.

### DELs may regulate NETosis-related genes in patients with SCAP

**Figure 4:**
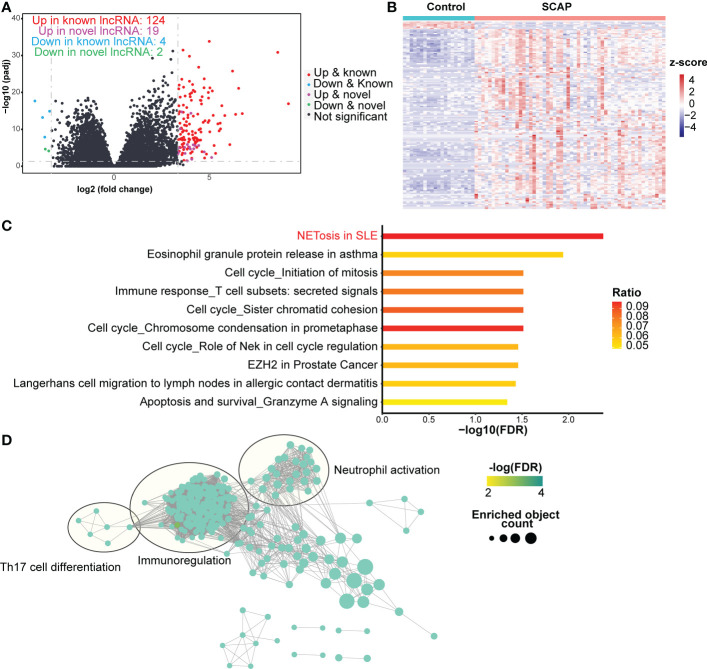
DELs identification and function prediction. **(A)** Volcano plot of lncRNAs. The horizontal dashed line represents the P-value cutoff (0.05) for differentially expressed lncRNAs (DELs). The vertical dashed line represents the fold change (FC) cutoff for DELs in patients relative to healthy controls (10 and 0.1). **(B)** Heatmap of DELs. **(C)** Pathway enrichment of trans target genes. The ratio indicates the proportion of the number of enriched network objects in a pathway to the total number of network objects in the pathway. **(D)** Gene Ontology Biological Processes enrichment map of trans target genes. The connection between biological processes is based on shared objects.

To understand the role of lncRNAs in patients with SCAP, we analyzed known and novel lncRNAs in our RNA-seq data and identified 152 significant DELs (FC > 10 for upregulated DELs or FC < 0.1 for down-regulated DELs, adjusted P-value < 0.05), including 124 upregulated known DELs, 19 upregulated novel DELs, four downregulated known DELS, and two downregulated novel DELs (**Figure 4A**). Patients with SCAP and healthy controls displayed different DEL expression patterns (**Figure 4B**).

Based on the two regulatory modes of lncRNAs (34), we predicted the target genes of the DELs in two ways. We clustered cis-acting and trans-acting target genes using the MetaCore website to determine the functions of the DELs. The NETosis pathway ranked first and sixth in the pathway enrichment analysis results for the trans-acting and cis-acting target genes, respectively (**Figure 4C**; **Supplementary Figure 1B**). This indicates that DELs may participate in NETosis by regulating NETosis-related protein-coding genes.

GO enrichment analysis revealed that both cis- and trans-acting target genes were involved in the biological processes of immunoregulation, neutrophil activation, and Th17 cell differentiation (**Figure 4D**; **Supplementary Figure 1C**). Only cis-acting target genes were involved in neutrophil-mediated killing (**Supplementary Figure 1B**). During NETosis, neutrophils release cathelicidin (35), a host defense peptide that promotes Th17 cell differentiation (36). This may be one explanation for the increased frequency of Th17 cells in the blood of patients with pneumonia (2, 3). Additionally, immunoregulation may be present in patients with SCAP as it can restrict excessive NETosis (37).

### Downstream genes of gene pairs with TRT are highly enriched in the NETosis pathway

**Figure 5:**
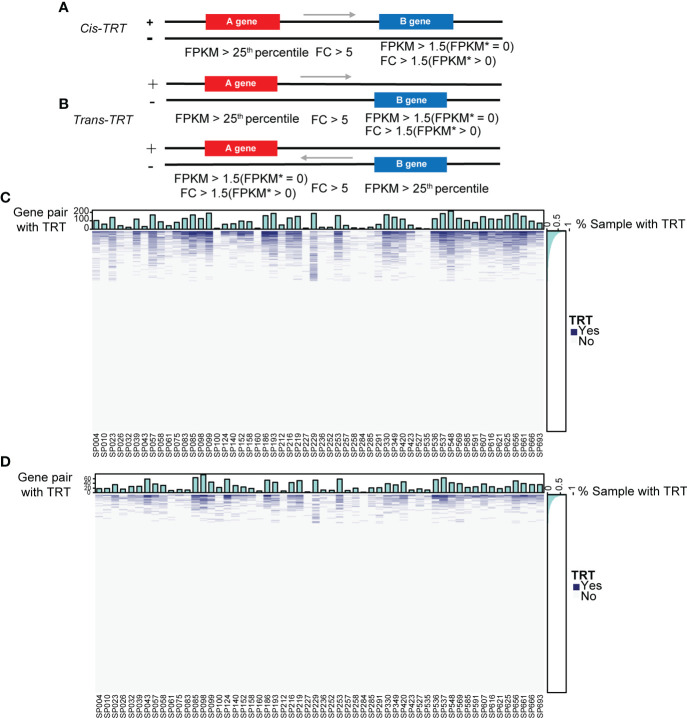
Overview of cis- and trans-TRT. **(A)** The cis-transcriptional read-through (cis-TRT) screening scheme with an arrow representing the direction of transcription. Patient gene expression is shown as fragments per kilobase of exon per million mapped fragments (FPKM). FPKM* represents the mean gene expression in healthy controls. **(B)** trans-TRT screening scheme. **(C)** A cis-TRT heatmap illustrating gene pairs with (blue) and without (grey) TRT. The top bar plot depicts the number of gene pairs with TRT per patient sample. The right bar plot indicates the percentage of samples in which TRT was observed for each gene pair. **(D)** trans-TRT heatmap.

Mounting evidence has shown that viral infections can induce TRT, especially influenza (13, 15, 16, 38). We wondered whether TRT could occur in patients with SCAP. To verify this, we selected all adjacent gene pairs in the genome, either on the same strand (cis-TRT) or different strands (trans-TRT), and determined whether these gene pairs underwent read-through transcription only in patients with SCAP by three-step screening (**Figures 5A, B**). Heatmaps and corresponding bar plots were used to illustrate the occurrence of patient-specific cis- and trans-TRT (**Figures 5C, D**).

**Figure 6:**
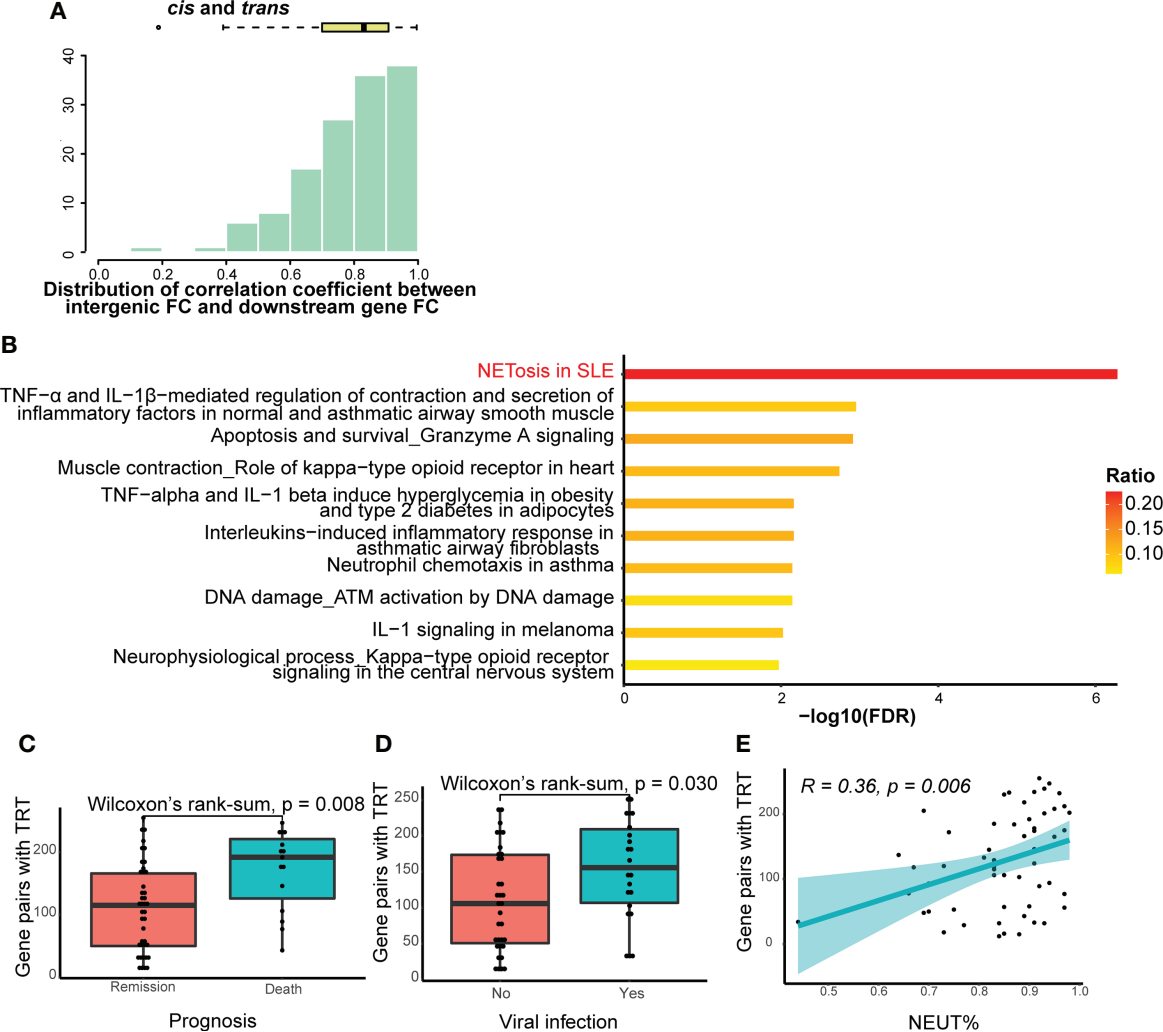
Further analysis of TRT. **(A)** Histogram and box-and-whisker plots depicting Spearman’s correlation between intergenic region fold change (FC) and downstream gene FC. Gene pairs with cis- or trans-TRT in more than 30% of patients with SCAP were selected for visualization. The lines in the box-and-whisker plots represent the median Spearman’s correlation coefficient and the 0.25 and 0.75 quantiles. **(B)** Pathway enrichment analysis of the downstream genes of gene pairs with cis- or trans-TRT. The ratio indicates the proportion of the number of enriched network objects in a pathway to the total number of network objects in the pathway. **(C)** The number of gene pairs with cis- or trans-TRT in patients with different prognoses. The lines in the box-and-whisker plots represent the median of the number of gene pairs with TRT and the 0.25 and 0.75 quantiles. Significance was determined using Wilcoxon rank-sum tests. **(D)** The number of gene pairs with cis- or trans-TRT in patients with or without viral infection. **(E)** Spearman’s correlation between the numbers of gene pairs with cis- or trans-TRT and the percentage of neutrophils. The light-colored area indicates the confidence interval.

For further analysis, we selected gene pairs with TRT in > 30% of patients with SCAP. First, we calculated the correlation coefficient between intergenic and downstream gene FC for each selected gene pair. Spearman’s correlation coefficients for cis-, trans-, and their combined total-TRT were positive and mainly within the 0.7−0.9 range (**Figure 6A**; **Supplementary Figures 2A**, **3A**), suggesting that the occurrence of TRT may increase downstream gene transcription in patients with SCAP. We then performed pathway enrichment analysis for downstream genes of the selected gene pairs in cis-, trans-, and total-TRT. The NETosis pathway was significantly enriched (**Figure 6B**; **Supplementary Figures 2B**, **3B**), especially in cis- and total-TRT. This indicates that the downstream genes affected by TRT are involved in NETosis.

We also analyzed the relationship between clinical traits and the number of gene pairs with cis-, trans-, or total-TRT. There was a significant difference in the number of gene pairs with TRT (P-value = 0.028 in cis-TRT, P-value = 0.0001 in trans-TRT, P-value = 0.0008 in total-TRT) between patients who died and those who went into remission (**Figure 6C**; **Supplementary Figure 2C**, **3C**). In addition, the patients were divided into two groups based on whether they were infected by viruses (**Table 1**). The number of gene pairs with TRT in patients with viral infections was significantly higher than in those without viral infections for trans- and total-TRT, but not cis-TRT (P-value = 0.02 in trans-TRT, P-value = 0.03 in total-TRT, P-value = 0.067 in cis-TRT; **Figure 6D**; **Supplementary Figures 2D**, **3D**). Additionally, there was no significant difference in prognosis between patients with or without viral infections (chi-square test: P-value = 0.073).

We then examined the correlation between the number of gene pairs with TRT and clinical traits. The only clinical trait significantly correlated with the number of gene pairs with cis-, trans-, and total-TRT was the percentage of neutrophils. (**Supplementary Figure 2E**, Spearman’s R = 0.31, P-value = 0.019 in cis-TRT; **Supplementary Figure 3E**, Spearman’s R = 0.46, P-value = 0.00032 in trans-TRT; **Figure 6E**, Spearman’s R = 0.36, P-value = 0.0058 in total-TRT).

### Potential therapeutic targets are identified in the NETosis pathway for patients with SCAP

**Figure 7:**
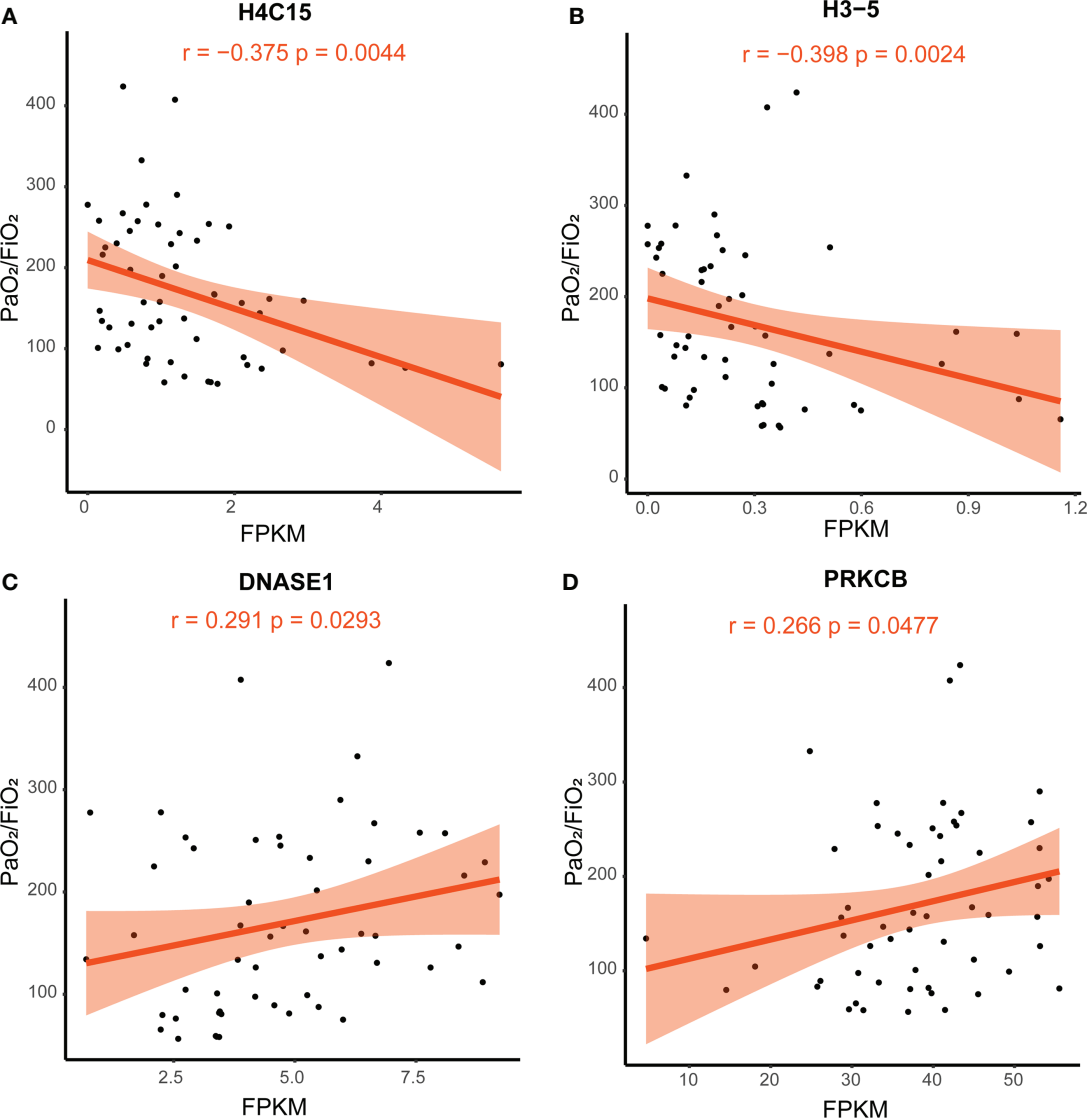
Correlation of PaO_2_/FiO_2_ and gene FPKM. Spearman’s correlation coefficient was used. The light-colored area indicates the confidence interval. **(A)** The correlation between the expression of H4C15 and PaO_2_/FiO_2_. **(B)** The correlation between the expression of H3-5 and PaO_2_/FiO_2_. **(C)** The correlation between the expression of DNASE1 and PaO_2_/FiO_2_. **(D)** The correlation between the expression of PRKCB and PaO_2_/FiO_2_. FPKM, fragments per kilobase of exon per million mapped fragments; FiO_2_, Fraction of inspired oxygen; PaO_2_, partial pressure of oxygen.

To identify potential therapeutic targets in patients with SCAP, we calculated Spearman’s correlation coefficient between the fragments per kilobase of exon per million mapped fragments (FPKM) of genes in the NETosis pathway and PaO_2_/FiO_2_. The expression levels of *H4C15* and *H3-5* were significantly negatively correlated with PaO_2_/FiO_2_ (Spearman’s R = −0.375, P-value = 0.0044; Spearman’s R = −0.398, P-value = 0.0024; **Figures 7A, B**), whereas the expression level of *DNASE1* and *PRKCB* were significantly positively correlated with PaO_2_/FiO_2_. (Spearman’s R = 0.291, P-value = 0.0293; Spearman’s R = 0.266, P-value = 0.0477; **Figures 7C, D**). These correlations suggest that patients with higher expression of *H4C15* and *H3-5*, and lower expression of *DNASE1* and *PRKCB*, are likely to suffer more severe lung injury.

## Discussion

In this study, we discovered that NETosis was top-ranked in pathway enrichment of differentially expressed genes, predicted target genes of differentially expressed long non-coding RNAs, and downstream genes of gene pairs with readthrough transcription in patients with SCAP caused by diverse kinds of pathogens. We identified DEGs and DELs between patients with SCAP and healthy controls. Pathway enrichment analyses of DEGs and the target genes of DLEs indicated a critical role for NETosis in SCAP. GO Biological Processes term enrichment analyses revealed that some biological processes involving both DEGs and the target genes of DELs overlap with known cellular mechanisms of NETosis (33, 35–37). We also identified gene pairs with patient-specific TRT in more than 30% of the patients with SCAP and found that the NETosis pathway was the most enriched pathway in the clustered downstream genes of the identified gene pairs. Previous studies in mouse models have shown that NETosis is implicated in pneumonia caused by influenza, *Klebsiella pneumoniae*, aspergillosis, and *Pseudomonas aeruginosa* (39–42). However, relevant researches in patients with severe pneumonia and studies about several pneumonia-causing pathogens are absent, such as adenovirus, *Pneumocystis carinii*, Mycoplasma, and cytomegalovirus. Sometimes, pathogens in severe pneumonia are difficult to identify and can be multiple, which limits the transfer of basic research on NETosis into the clinic. Our work goes beyond previous studies, suggesting that NETosis play a critical role in diverse kinds of severe pneumonia and viral infection may induce more active NETosis (**Figure 3A**). This provides a potential therapeutic strategy to treat patients by targeting NETosis even though pathogens are unknown or multiple.

Like all processes, excessive NETosis can be detrimental to host. Several studies have shown elevated circulating NET components in sepsis are associated with multi-organ failure and poor prognosis (43–45). As the released components in NET is non-specific, NET can cause cell damage and organ injury directly (46, 47). In addition, NETs can recruit inflammatory cells and substances, serve as a platform for complement activation, induce the production of autoantibody, promote the formation of immune complexes, and promote vascular occlusion (48–50), thus leading to tissue damage. Our results also suggested that patients with more active NETosis were more prone to acute kidney injury and more severe lung injury.

Furthermore, we used the PaO_2_/FiO_2_ ratio to represent the severity of lung injury and explored potential therapeutic targets for NETosis. There was a significant negative correlation between *H4C15* and *H3-5* expression and PaO_2_/FiO_2_ and a significant positive correlation between *DNASE1* and *PRKCB* expression and PaO_2_/FiO_2_. In NETosis, histone posttranslational modifications, such as citrullination by peptidyl arginine deiminase 4 (PAD4) and acetylation, mediate chromatin decondensation that characterizes NETosis compared with other cell death processes (33). Moreover, histone is an essential component of NET. Higher expression levels of histone suggest more active NETosis, resulting in severer lung injury and lower PaO2/FiO2. The differences in the role of different histones in the NETosis process remain unclear. The differences may relate to post-transcriptional modifications of histones, such that H3 possesses more sites that can be modified by PAD4 (33). Protein kinase C (PKC) is a crucial mediator in NETosis. The PKC inhibitor Gö6976 can block the NET formation in NETosis induced by phorbol 12-myristate 13-acetate, *Candida albicans*, and Group B Streptococcus (51). However, a study showed PKCα and PKCβ could repress histone citrullination, whereas PKCζ could activate PAD4 and then facilitate NETosis. The correlation between PRKCB expression and PaO_2_/FiO_2_ is challenging to interpret due to the previous inconsistent findings. More research on the mechanism of NETosis is needed. DNase I mediate the clearance of NETs (52). Higher expression levels of DNase I suggest more NET degradation, leading to slighter lung injury and higher PaO2/FiO2. In venom-induced NETosis, DNase 1 treatment can prevent or reverse NET formation, thus protecting the tissue from NET-mediated destruction (53). Likewise, long-acting nanoparticulate DNase 1 inhibits NETosis in the plasma of patients with COVID-19 and a septic mouse model (54). These studies suggest that *DNASE1* possesses more potential to become a therapeutic target for NETosis.

Another important finding of our study concerns TRT. We analyzed the relationship between the number of gene pairs with patient-specific TRT and clinical traits. TRT is more likely to occur in patients with viral infections, consistent with previous studies, which demonstrated that TRT could be induced by HSV-1 (13) and influenza (15, 16, 38). Our results also suggest that other viral infections (such as cytomegalovirus, adenovirus, and Epstein–Barr virus) and even bacteria and fungi may induce TRT. The exact function of read-through transcripts remains unclear. Our study found that patients with more gene pairs undergoing TRT tended to have a worse prognosis, suggesting that read-through transcripts may be a by-product of transcription under diverse cellular stresses. TRT may represent an imbalance in cellular homeostasis. This provides a novel perspective for explaining the high mortality rate of patients with SCAP.

In this study, some limitations need to be considered. Patients were significantly older than healthy controls (P < 0.001; **Supplementary Table 5**). Some studies have shown that Aging in humans and mice impairs the formation of NETs (55, 56). However, all genes enriched in the NETosis pathway were upregulated in our study (**Supplementary Table 6**, **7**), indicating active NETosis and increased NET formation. And there was no significant correlation between NETosis and patient age in this study (**Supplementary Table 4**). Therefore, our conclusions are not affected by this limitation. Moreover, using bulk transcriptome data of peripheral blood leukocytes to study NETosis may introduce confounding factors because high neutrophil percentages in the SCAP group may affect the sequence coverage of NETosis-related genes. We acknowledge that this limitation may cause bias, so we try to reduce the bias by identifying DEGs and DELs with a large fold change. In the future, we will conduct further research by using Single-cell RNA sequencing to investigate the role of neutrophils in patients with SCAP.

In conclusion, we discover that NETosis is critical in SCAP and highlight *H4C15*, *H3-5*, *DNASE1*, and *PRKCB* as promising therapeutic targets for severe pneumonia. Our data also contribute to the current understanding of TRT, as they demonstrate that 1) viral infection is more likely to induce TRT (although other pathogens may also induce TRT) and 2) a higher occurrence of TRT is associated with a worse outcome in patients with SCAP.

## Data availability statement

The RNA-seq data obtained in this study have been deposited in NCBI's Gene Expression Omnibus and are available through accession number GSE196399, https://www.ncbi.nlm.nih.gov/geo/query/acc.cgi?acc=GSE196399.

## Ethics statement

This study was reviewed and approved by the Ethics Committee of the Peking Union Medical College (S500). Written informed consent was obtained from all individuals.

## Author contributions

YZ, CJ, and JX designed the study. YZ and NS analyzed the data. YZ, YLi, HT, YLiu, YF, HZ, CJ, and JX interpreted the data. YZ, NS, and YLi wrote the manuscript. CJ and JX edited the manuscript. JY and QH collected the data. Authors have read and approved the manuscript.

## Funding

This research was supported by the National Natural Science Foundation of China (81788101, 32100104), the Chinese Academy of Medical Sciences Innovation Fund for Medical Sciences (2021–I2M-1–022, 2021-I2M-1-062), the 111 Project (BP0820029), and the CAMS Endowment Fund (2021-CAMS-JZ001). The funders had no role in the study design, data collection, data analysis, data interpretation, and manuscript writing.

## Acknowledgments

We thank all patients and control participants. We thank Song Mei and Yexuan Lin for their advice on transcriptome analyses.

## Conflict of interest

The authors declare that the research was conducted in the absence of any commercial or financial relationships that could be construed as a potential conflict of interest.

## Publisher’s note

All claims expressed in this article are solely those of the authors and do not necessarily represent those of their affiliated organizations, or those of the publisher, the editors and the reviewers. Any product that may be evaluated in this article, or claim that may be made by its manufacturer, is not guaranteed or endorsed by the publisher.
