# Supplementary figures and images for: NETosis is critical in patients with severe community-acquired pneumonia
Source: Front Immunol. 2022 Nov 15;13:1051140. doi: 10.3389/fimmu.2022.1051140 (PMC9709478; doi:10.3389/fimmu.2022.1051140)

# Supplemental figure 1

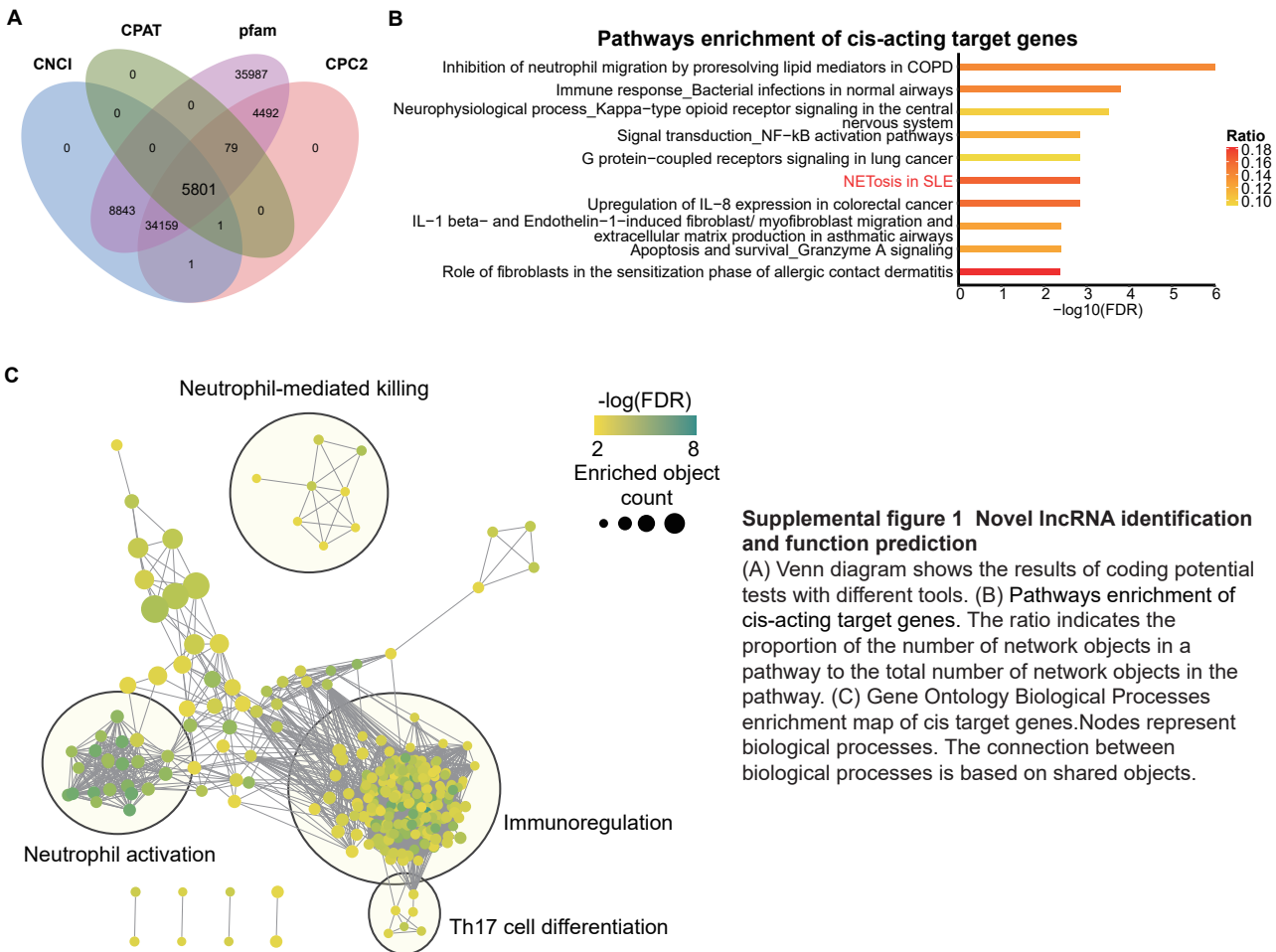

Supplement: Supplementary file 1 [file Image_1.pdf]
