# Supplementary material for: NETosis is critical in patients with severe community-acquired pneumonia
Source: Front Immunol. 2022 Nov 15;13:1051140. doi: 10.3389/fimmu.2022.1051140 (PMC9709478; doi:10.3389/fimmu.2022.1051140)
Supplement: Supplementary file 3 [file Image_3.pdf]

Supplemental figure 3

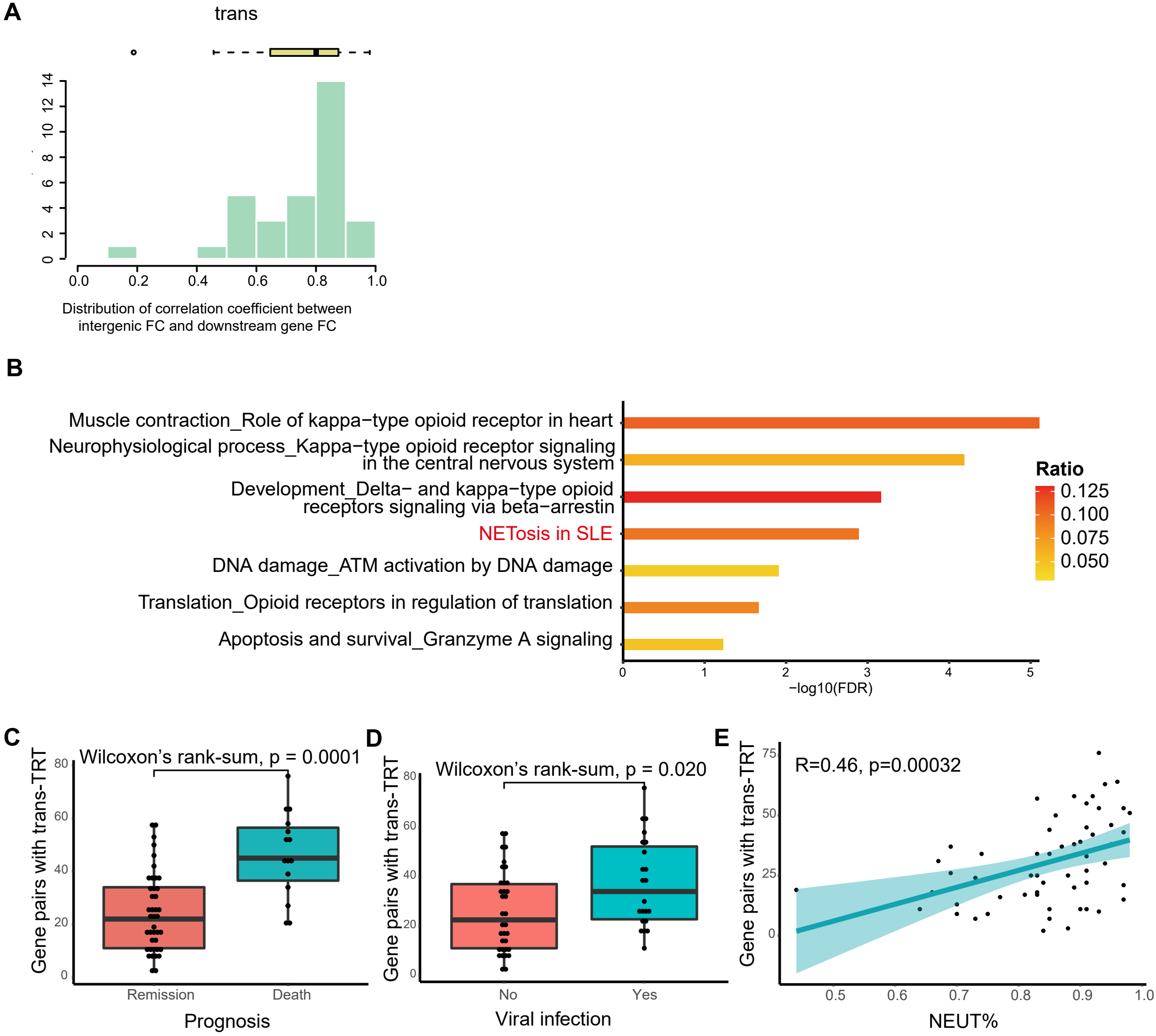

Supplemental figure 3 Further analysis of trans-TRT

(A) Histogram and box-and-whisker plots depicting Spearman's correlation between intergenic region fold change (FC) and downstream gene FC. Gene pairs with trans-TRT in more than 30% of patients with SCAP were selected for visualization. The lines in the box-and-whisker plots represent the median Spearman's correlation coefficient and the 0·25 and 0·75 quantiles. (B) Pathway enrichment analysis of the downstream genes of gene pairs with trans-TRT > 30% of patients with SCAP. The ratio indicates the proportion of the number of enriched network objects in a pathway to the total number of network objects in the pathway. (C) The number of gene pairs with trans-TRT in patients with different prognoses. The lines in the box-and-whisker plots represent the median of the number of gene pairs with TRT and the 0·25 and 0·75 quantiles. Significance was determined using Wilcoxon rank-sum tests. (D) The number of gene pairs with trans-TRT in patients with or without viral infection. (E) Spearman's correlation between the numbers of gene pairs with trans-TRT and the percentage of neutrophils. The light-colored area indicates the confidence interval.
